# Modeling the *α*-max capacity of transportation networks: a single-level mathematical programming formulation

**DOI:** 10.1007/s11116-021-10208-1

**Published:** 2021-07-14

**Authors:** Zhaoqi Zang, Xiangdong Xu, Anthony Chen, Chao Yang

**Affiliations:** 1 Key Laboratory of Road and Traffic Engineering of the Ministry of Education, Tongji University, Shanghai, China; 2 Department of Civil and Environmental Engineering, The Hong Kong Polytechnic University, Kowloon, Hong Kong China

**Keywords:** Network capacity, Trip level of service, Flexibility, Convex programming, Gradient projection

## Abstract

Network capacity, defined as the largest sum of origin–destination (O–D) flows that can be accommodated by the network based on link performance function and traffic equilibrium assignment, is a critical indicator of network-wide performance assessment in transportation planning and management. The typical modeling rationale of estimating network capacity is to formulate it as a mathematical programming (MP), and there are two main approaches: single-level MP formulation and bi-level programming (BLP) formulation. Although single-level MP is readily solvable, it treats the transportation network as a physical network without considering level of service (LOS). Albeit BLP explicitly models the capacity and link LOS, solving BLP in large-scale networks is challenging due to its non-convexity. Moreover, the inconsideration of trip LOS makes the existing models difficult to differentiate network capacity under various traffic states and to capture the impact of emerging trip-oriented technologies. Therefore, this paper proposes the *α*-max capacity model to estimate the maximum network capacity under trip or O–D LOS requirement *α*. The proposed model improves the existing models on three aspects: (a) it considers trip LOS, which can flexibly estimate the network capacity ranging from zero to the physical capacity including reserve, practical and ultimate capacities; (b) trip LOS can intuitively reflect users’ maximum acceptable O–D travel time or planners’ requirement of O–D travel time; and (c) it is a convex and tractable single-level MP. For practical use, we develop a modified gradient projection solution algorithm with soft constraint technique, and provide methods to obtain discrete trip LOS and network capacity under representative traffic states. Numerical examples are presented to demonstrate the features of the proposed model as well as the solution algorithm.

## Introduction

As a critical indicator of network-wide performance assessment, network capacity has been used in many transportation problems such as traffic control (Wong and Yang 1997; Ceylan and Bell 2004; Chiou 2014), road pricing (Xu et al. 2017; Yang and Huang 2005), demand manage scheme (Akamatsu and Wada 2017), land use optimization (Yim et al. 2011), car ownership estimation (Tam and Lam 2000), network design (Yang and Bell 1998; Lo and Tung 2003; Chen et al. 2011a; Miandoabchi and Farahani 2011), and network assessment like capacity reliability (Chen et al. 2002), capacity flexibility (Chen and Kasikitwiwat 2011), network vulnerability (Bell et al. 2017; Xu et al. 2018a), and network redundancy (Xu et al. 2018b), etc. However, evaluating the network-wide capacity is a nontrivial task since it is not just a simple arithmetic operation (e.g., min, max, mean) of the individual link capacities in the network. Different from the classical maximum network flow problem in graph theory, measuring transportation network capacity has a few challenges. There are multiple origin–destination (O–D) pairs and their travel demands are not exchangeable or substitutable. Also, various boundary and behavioral constraints restrain the network capacity. Typical examples of the boundary constraints are link capacity constraints and zonal trip production and attraction constraints that restrain the capacity of the whole transportation network. For the behavioral constraints, individual users’ travel choice behaviors (e.g., destination choice, mode choice, route choice, etc.) and collective congestion effect should be captured in estimating the network capacity. *These boundary and behavioral constraints pose great challenges to the modeling of transportation network capacity.*

### Methodologies and challenges of modeling the network capacity

In the literature, there are two main modeling methodologies for estimating the transportation network capacity: single-level mathematical programming (MP) formulation and bi-level programming (BLP) formulation. For the single-level MP formulation, Akamatsu and Miyawaki (1995) adopted the excess demand formulation (Sheffi 1985) to calculate the equilibrium network capacity by using an augmented network representation. By setting travel time on the dummy link between each O–D pair as a large enough constant, an approximate value of the maximum network flow can be obtained by solving a single-level fixed-demand user equilibrium (UE) traffic assignment problem (TAP). The single-level MP formulation makes this model readily solvable with convergent algorithms and global optimum. Due to the necessity of considering travelers’ choice behaviors, many existing studies formulated the network capacity problem as a BLP. For instance, Asakura (1992) formulated the equilibrium network capacity problem with a prescribed O–D pattern as a bi-level optimization problem. For a signal-controlled road network, Wong and Yang (1997) proposed the concept of *reserve capacity* to seek an optimal signal control pattern. Gao and Song (2002) extended the reserve capacity model of Wong and Yang (1997) to consider O–D pair-specific demand multipliers.

However, as commented by Yang et al. (2000), there are two key issues of the prior works: (1) the network capacity defined in Akamatsu and Miyawaki (1995) is actually treated as a maximum physical amount of flow capable of being accommodated (i.e., the physical network capacity), and is not related to the level of service (LOS) of road network; (2) the reserve capacity model needs to assume a target O–D matrix or a current trip demand pattern. To this end, Yang et al. (2000) formulated the network capacity and LOS problem as a BLP with the upper level to maximize the total zonal trip production subject to the lower level as a combined trip distribution and assignment problem, which allows both destination choice and route choice without the need to assume the target O–D matrix. Chen and Kasikitwiwat (2011) and Chen et al. (2013) further detailed the network capacity model of Yang et al. (2000) as the ultimate and practical network capacity models in assessing the capacity flexibility and capacity reliability of transportation networks.

Again, these studies formulated the network capacity problem as a BLP, where the lower-level captures the users’ travel choice behaviors (i.e., route choice for the reserve capacity model, and both destination and route choices for the practical and ultimate network capacity models), and the upper-level maximizes different forms of total network throughput (i.e., a single multiplier for the reserve capacity model, and total trip production for the network capacity and LOS problem). Although BLP can explicitly model the hierarchical game between leaders and followers, it is generally non-convex, leading to undesirable properties (e.g., non-uniqueness and local optimality). *Consequently, it is quite challenging to develop a computationally efficient algorithm of BLP for large-scale network applications* despite that lots of solution algorithms (e.g., Wang and Lo 2010; Luathep et al. 2011; Farvaresh and Sepehri 2013; Possel et al. 2018) have been developed.

Another challenge is the necessity of considering *trip or O–D-based LOS* in network capacity modeling. The LOS in the existing BLP models is generally considered as *link-based*. In other words, the LOS is defined as the requirement that the flow on each link is less than or equal to its capacity, or the maximum volume-to-capacity (V/C) ratio of each link should be below a prescribed value (e.g., Wong and Yang 1997; Yang et al. 2000; Gao and Song 2002; Chen and Kasikitwiwat 2011; Chen et al. 2013; Xu et al. 2018b). However, for users, they pay more or direct attention to the O–D travel time (or cost) rather than the operating conditions of a single link. The O–D travel time is the price paid for fulfilling the purpose of reaching the destination. As a result, the value of O–D travel time can significantly affect their trip choices such as to travel or not (i.e., trip generation), when to travel (i.e., departure time choice), and which mode to use (i.e., mode choice). Recently emerged user- or trip- oriented technologies and services such as mobility as a service (MaaS) further highlight the importance of trip LOS. Besides, planners also attach more importance to O–D-based LOS and even set O–D-based LOS as planning targets. For example, the Shanghai City Master Plan for 2035 wants to ensure that the average commuting time in central city is less than 40 min (Shanghai 2017), while New York plans to provide 90% of New Yorkers with access to more than 200,000 jobs by transit in 45 min (New York 2015). Therefore, to address the above concerns of both users and planners, *it is necessary to explicitly consider the O–D-based LOS when modeling the network capacity*.

The last challenge is about the deep understanding of network capacity under various traffic conditions, which is an important basis for enacting meticulous traffic management measures. For example, to contain the spread of COVID-19, strict lockdown is adopted by many countries (Chinazzi et al. 2020; Lau et al. 2020; Zhang et al. 2020), but it also leads to devastating economic consequences. It may be better to use cyclic work-lockdown strategy (Karin et al. 2020) or some soft interventions (Koh et al. 2009; Zhang and Qian 2019; Wang et al. 2020) to replace strict lockdown, for providing (though reduced) sustainable economy. Specifically, we can lower the network capacity of transportation network to contain the spread of COVID-19 when there are more cases. If observing a strong decreasing trend of COVID-19, we may increase the network capacity to gain more economic benefits. This requires a deep understanding of network capacity under various traffic conditions to help designing or enacting various possible management measures. Nevertheless, the existing network capacity models cannot provide this flexibility to estimate the value of network capacity in such a wide range.

Note that in the literature, there is another line of approach to estimate the network capacity based on the macroscopic fundamental diagram (MFD). Compared to the MFD approach, the approach in the above discussion can be referred to as mathematical programming (MP)-based approach, which concerns the development of MP formulation to estimate the network capacity. Table 1 summarizes these two approaches with respect to theoretical foundations, definitions of capacity, impact factors, application, and representative literature.

**Table 1:** A summary of the MP-based approach and the MFD approach for estimating the network capacity

Dimensions	MP-based approach	MFD
Theoretical foundations	Link performance function (e.g., the well-known Bureau of Public Roads function) and traffic equilibrium assignment	Macroscopic or network fundamental diagram
Definition of capacity	The largest sum of O–D flows that can be handled by the network	The maximum network flow on the (theoretical) network FD
Impact factor	Parking restriction, target O–D pattern, link capacity constraint, route choice behavior, etc	Topology, traffic signals, link lengths, offsets, spatial distribution of congestion, etc
Application	Car ownership estimation, network assessment, network design, traffic signal control, road pricing, network reliability, network flexibility, network redundancy, etc	Dynamic traffic management and control, road pricing, etc
Literature	Iida (1972), Asakura (1992), Asakura and Kashiwadani (1993), Akamastu and Miyawaki (1995), Wong and Yang (1997), Yang et al. (2000), Chen et al. (2002), Gao and Song (2002), Chen and Kasikitwiwat (2011), Chen et al. (2013), Wang et al. (2018), Xu et al. (2018a, b)	Mazloumian et al. (2010), Boyacı and Geroliminis (2011), Geroliminis and Boyacı (2012), Yildirimoglu and Geroliminis (2014), Saberi et al. (2015)

**Fig. 1:**
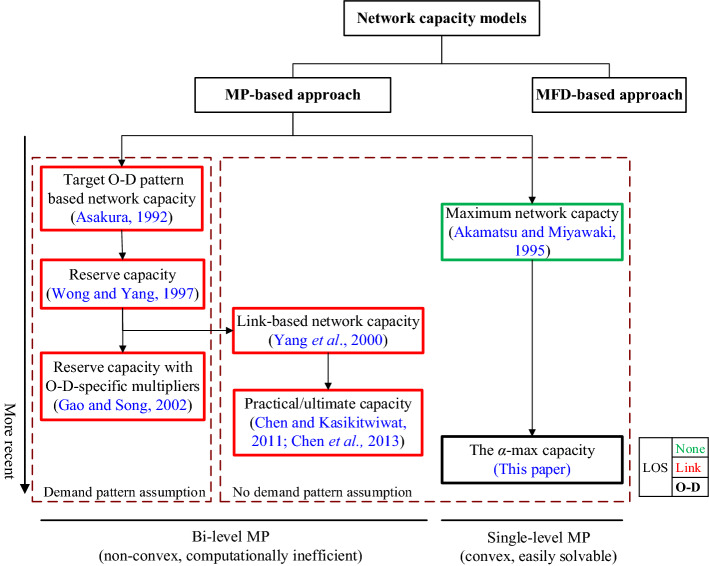
Chronicle development and classification of MP-based capacity models

Based on Table 1, we can conclude that the theoretical foundations, the focus of definition of network capacity, and the impact factors of these two approaches are different. Besides, there are both similarities and differences for the application of these two approaches. On the one hand, the applications of the MP-based approach focus on the long-term evaluation of network performance and network design, while the applications of the MFD approach consider the short-term assessment of dynamic traffic management and control. On the other hand, both can be used for traffic control and road pricing for different planning horizons (e.g., strategic planning versus operation planning). It should be noted that this paper focuses on the development of a single-level MP formulation for estimating the network capacity with trip level of service, which belongs to the MP-based approach. Figure 1 provides a brief summary and classification of MP-based network capacity models with respect to the MP formulation, the demand pattern assumption, and the LOS consideration.

### Main contributions of this paper

To fill the research gap, this paper proposes the *α*-max capacity model by formulating the network capacity with the trip LOS consideration as a single-level MP. The proposed *α*-max capacity model estimates the maximum network capacity according to the requirement of O–D-based LOS *α*. Specifically, using the concept of super-network, this model constructs an augmented network representation to formulate the network capacity model as a generalized excess-demand UE formulation. For each O–D pair in the augmented network, we consider a virtual path with the cost being equal to *α·*times of the minimum free-flow path cost *τ*_min_ of this O–D pair, which can be interpreted as the maximum O–D cost acceptable for users under the O–D-based LOS requirement *α*. Thus in our model, when the actual O–D cost is less than the maximum acceptable O–D cost *ατ*_min_, users are “pushed” as much as possible to the physical network, which in turn results in the increase of the actual O–D cost. Therefore, the maximum absorbable O–D flow *q* in the physical paths for each O–D pair can be obtained when actual O–D cost cannot increase any more (i.e., less than or equal to *ατ*_min_). This allows two choices: one choice for deciding whether to travel or not subject to a required O–D-based LOS, and another choice for route selection according to the UE criterion. Besides, different *α* values would correspond to different maximum acceptable O–D costs and thus different network capacity values, which offers the proposed model great flexibility to model network capacity under various conditions. We further provide the lower and upper bounds of the network capacity derived from the *α*-max capacity model.

The above corresponding relationship between LOS requirements and network capacity values can be analogous to the concept of maximum flow rate for each LOS class in the Highway Capacity Manual (TRB 2010). Therefore, a general framework and machine learning methods are provided for deriving the thresholds of *α* to classify trip LOS into different LOS categories and for obtaining *α* values under representative traffic states. To solve the proposed *α*-max capacity model, a modified gradient projection algorithm with soft constraint technique is developed. The soft constraint technique always guarantees the feasible solution because the penalization of violating side constraints is imposed in the objective function without destroying the Cartesian product structure of the feasible set (Patriksson 2004, 2015; Nie et al. 2004). Numerical examples using an illustrative network and a realistic large-scale network are also presented to demonstrate the features of the proposed model as well as the applicability of the solution algorithm for large-scale network applications.

In summary, compared to the existing network capacity models, the features of the proposed model are threefold: (a) it considers trip LOS, which offers great flexibility to estimate the network capacity ranging from zero^1^ to the physical capacity including reserve, practical and ultimate capacities; (b) trip LOS can intuitively reflect users’ maximum acceptable O–D travel cost and also answer planners’ question about what is the network capacity under a certain O–D-based LOS requirement; and (c) it is a convex and tractable single-level MP, which is convenient for developing a computationally efficient solution algorithm for large-scale network applications.

The remainder of this paper is organized as follows. Section “Illustration of the modeling idea” illustrates our modeling idea of network capacity. The proposed *α*-max capacity model and discussions of its properties are provided in “The *α*-max network capacity model” section. Section “Solution algorithm” presents the solution algorithm. Numerical examples and conclusions are summarized in “Numerical examples” and “Concluding remarks” sections, respectively.

## Illustration of the modeling idea

**Fig. 2:**
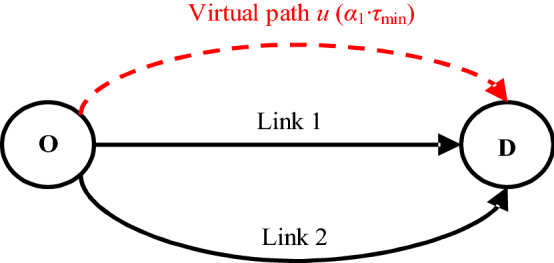
A simple network example modified from Yang et al. (2000)

**Fig. 3:**
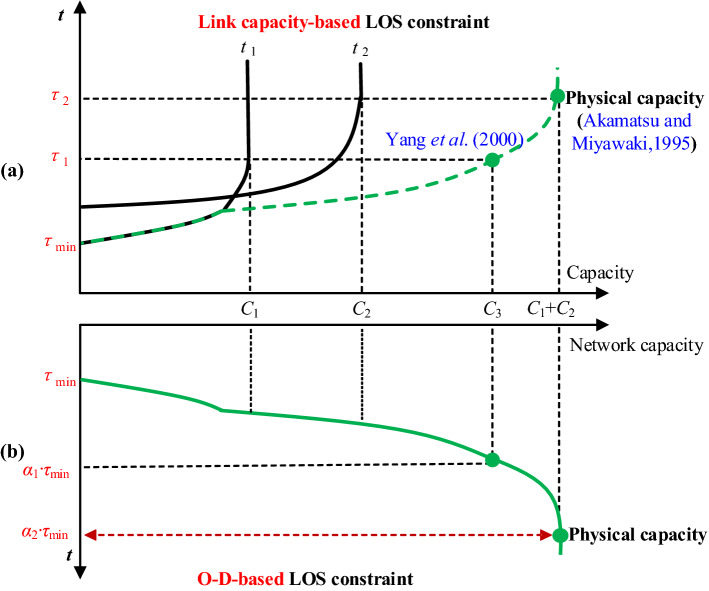
The relationship between the network capacity and the travel time of the two real links (**a**) and the virtual path (**b**)

Similar to Yang et al. (2000), a simple network with two real links and a virtual path as shown in Fig. 2 is considered. The travel time of each real link is assumed to increase vertically if its flow reaches its capacity. Figure 3 plots the relationship between the network capacity and the travel time of the two real links and the virtual path. *t*_1_, *t*_2_, and *u* denote the travel time of link 1, link 2, and virtual path, respectively. *t*_1_ = *τ*_1_ and *t*_2_ = *τ*_2_ when the flows of link 1 and link 2 equal their capacities *C*_1_ and *C*_2_, respectively. *C*_3_ is the O–D flow when *t*_1_ = *t*_2_ = *τ*_1_. For illustration of our modeling idea, the network capacity model proposed by Akamatsu and Miyawaki (1995) is used to illustrate a different meaning of the virtual path travel time, while the network capacity model proposed by Yang et al. (2000) is used to illustrate a different way to consider the LOS and its benefit in modeling the network capacity.

As discussed in the Introduction, by setting a large enough constant of *u*, Akamatsu and Miyawaki (1995) calculated the maximum network capacity via solving a single-level fixed-demand UE TAP. Accordingly, for this simple network, *u* must be greater than or equal to *τ*_2_ to obtain the maximum network capacity *C*_1_ + *C*_2_, and the resultant equilibrium link flows are *v*_1_ = *C*_1_ and *v*_2_ = *C*_2_. As commented by Yang et al. (2000), the network capacity in Akamatsu and Miyawaki (1995) is actually treated as the maximum physical capacity (i.e., *C*_1_ + *C*_2_) and is not related to the LOS of the network. Instead of setting a large enough constant of *u*, in our *α*-max capacity model, *α·τ*_min_ replaces *u* and it is used to represent the *maximum acceptable O–D cost that users are willing to pay* for travel under a given LOS requirement *α*. The value of *α* represents the LOS requirement and *τ*_min_ is the free-flow travel time of the shortest path between this O–D pair. A larger *α* means a lower LOS requirement as well as a higher tolerance of accepting a longer travel time for a trip. Several special cases of *α* (i.e., *α* = 1, *α*_1_ and *α*_2_) shown in Fig. 3b can be used to further explain its physical meaning:

- *α* ≤ 1 means that no one is willing to pay for a trip, so the network capacity is zero;
- *α* = *α*_1_ means the maximum acceptable O–D cost that a user is willing to pay for a trip is *α*_1_*·τ*_min_ (i.e., *τ*_1_), and its corresponding network capacity (i.e., *C*_3_) is also the value of network capacity obtained by Yang et al. (2000) to be discussed in detail in next paragraph;
- *α* ≥ *α*_2_ means that a user is willing to pay any price as long as he/she can make a trip, so the network capacity is equal to the physical capacity of this simple network.

For the capacity model in Yang et al. (2000), the LOS is considered as link-based, and the network capacity problem therein is formulated as a BLP while ensuring no queuing delay of any link under the UE criterion. Accordingly, for this simple network, the capacity constraint of link 1 determines that the maximum O–D travel time cannot exceed *τ*_1_. Thus, the network capacity is *C*_3_ and the resultant equilibrium link flows are *v*_1_ = *C*_1_ and *v*_2_ = *C*_3_–*C*_1_. Different from the link-based LOS requirement of the BLP model in Yang et al. (2000), the LOS requirement in the proposed *α*-max capacity model is O–D based. Therefore, in this paper, *transportation network capacity is defined as the maximum capacity that the network can accommodate at a required O–D-based LOS ****α***. Different *α* values would correspond to different maximum acceptable O–D costs, which offers the proposed model great flexibility to model the network capacity under various conditions. Specifically, we can model the network capacity from zero to physical capacity, including the network capacities obtained by Yang et al. (2000) and Akamatsu and Miyawaki (1995) as two special cases. Due to this flexibility, we can model the network capacity according to the O–D LOS required by traffic planners and managers to accommodate future traffic growth. The modeling results, including network capacity, link flows and O–D demands, can provide valuable information for planners and managers to take more meticulous and effective management and control.

## The *α*-max network capacity model

In this section, we formulate the *α*-max capacity problem as a single-level MP, followed by some discussions of its properties.

### Mathematical formulation

**Fig. 4:**
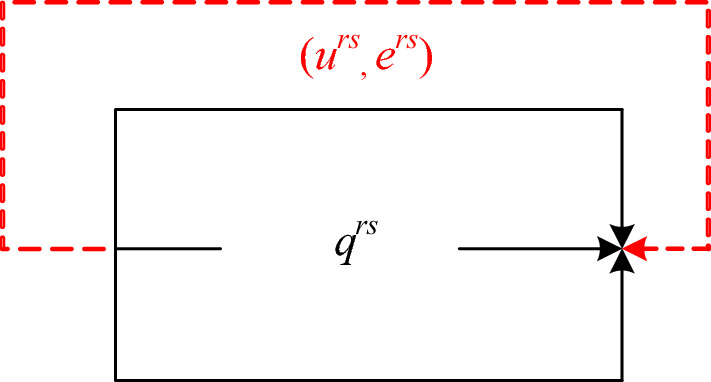
Excess-demand network representation for O–D pair (*r*, *s*)

Recall that in the augmented network shown in Fig. 4, the virtual path cost *u*^*rs*^ represents the maximum acceptable O–D cost that users are willing to pay for travel at required O–D-based LOS *α*. When the actual O–D cost is less than the maximum acceptable O–D cost *u*^*rs*^, users are “pushed” as much as possible to the physical network, which in turn results in the increase of the actual O–D cost. Therefore, the maximum absorbable O–D flow *q*^*rs*^ in the physical paths for each O–D pair can be obtained when the actual equilibrium O–D cost cannot increase any more (i.e., less than or equal to *u*^*rs*^). With this rationale, the virtual path flow can thus be interpreted as the excess (or unrealized) demand *e*^*rs*^ of exceeding the maximum absorbable demand *q*^*rs*^.

Therefore, the MP formulation of the *α*-max network capacity model is given by:

1 $$\min \;Z\left( {{\mathbf{f}},\;{\mathbf{q}}} \right) = \sum\limits_{{a \in A}} {\int_{0}^{{v_{a} }} {t_{a} \left( \omega \right)d\omega } } + \sum\limits_{{r \in R}} {\sum\limits_{{s \in S}} {u^{{rs}} e^{{rs}} } }$$

subject to

2 $$\sum\limits_{{k \in K^{{rs}} }} {f_{k}^{{rs}} } = q^{{rs}} ,\;\;\forall r \in R,\;s \in S$$

3 $$\sum\limits_{{r \in R}} {\sum\limits_{{s \in S}} {\sum\limits_{{k \in K^{{rs}} }} {f_{k}^{{rs}} \delta _{{ka}}^{{rs}} } } } = v_{a} ,\;\;\forall a \in A$$

4 $$q^{{rs}} + e^{{rs}} = \bar{Q}^{{rs}} ,\;\;\forall r \in R,\;s \in S$$

5 $$\sum\limits_{{s \in S}} {q^{{rs}} } \le \bar{O}^{r} ,\;\;\forall r \in R$$

6 $$\sum\limits_{{r \in R}} {q^{{rs}} } \le \bar{D}^{s} ,\;\;\forall s \in S$$

7 $$v_{a} \le C_{a} ,\;\;\forall a \in A$$

8 $$f_{k}^{{rs}} ,\;q^{{rs}} ,\;e^{{rs}} \ge 0,\;\;\forall k \in K^{{rs}} ,\;r \in R,\;s \in S$$

where *A* is the set of directed links in the network; *R* and *S* are the sets of origins and destinations; *K*^*rs*^ is the set of paths connecting O–D pair (*r*, *s*); *v*_*a*_, *t*_*a*_, and *C*_*a*_ are traffic flow, travel time, and capacity of link *a*, respectively; *f*_*k*_^*rs*^ is the flow on path *k* between O–D pair (*r*, *s*) and **f** is its vector form; $\delta _{{ka}}^{{rs}}$ is the link-path incidence indicator: $\delta _{{ka}}^{{rs}}$ = 1 if link *a* is on path *k* between O–D pair (*r*, *s*), and 0 otherwise; *q*^*rs*^ (**q** for vector form), *e*^*rs*^, and $\bar{Q}^{{rs}}$ are the realized demand, excess (i.e., unrealized) demand, and maximum potential demand between O–D pair (*r*, *s*); $\bar{O}^{r}$ and $\bar{D}^{s}$ are the maximum trip production at origin *r* and the maximum trip attraction at destination *s*.

Equation (2) is the incidence relationship that expresses realized O–D demands in terms of path flows; Eq. (3) is the incidence relationship that expresses link flows in terms of path flows; Eq. (4) is the travel demand conservation constraint between realized and excess demands, where we treat path flows and travel demands as explicit variables because the excess demands can be obtained from the travel demands; Eqs. (5) and (6) are the maximum trip production and trip attraction constraints, respectively; Eq. (7) is the link capacity constraint; Eq. (8) is the non-negativity constraint on path flows, and realized and excess demands.

**Fig. 5:**
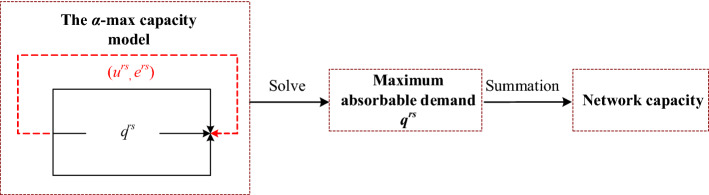
The modeling flowchart of estimating the network capacity in this paper

The maximum acceptable O–D cost *u*^*rs*^ in the objective function is defined as

9 $$u^{{rs}} = \alpha ^{{rs}} \cdot \tau _{{\min }}^{{rs}} ,\quad \forall r \in R,\;s \in S$$

where *α*^*rs*^ is the O–D-specific constant value of representing the required LOS between O–D pair (*r*, *s*). Specifically, $\tau _{{\min }}^{{rs}} = \mathop {\min }\nolimits_{{k \in K^{{rs}} }} \left\{ {\tau _{k}^{{rs}} } \right\}$ and $\tau _{k}^{{rs}}$ is the free-flow travel time of path *k* between O–D pair (*r*, *s*), i.e., $\tau _{k}^{{rs}} = \sum\nolimits_{{a \in A}} {t_{a}^{0} \delta _{{ka}}^{{rs}} }$ where *t*_*a*_^0^ is the free-flow travel time of link *a*. Figure 5 shows our modeling flowchart of estimating the network capacity. That is to say, through solving the *α*-max network capacity model, we can obtain the assigned O–D flows (i.e., maximum absorbable O–D flow *q*^*rs*^ in the physical paths for each O–D pair). Then the network capacity $\phi$ of transportation network can be obtained by the summation of all assigned O–D flows:

10 $$\phi = \sum\limits_{{r \in R}} {\sum\limits_{{s \in S}} {q^{{rs}} } }$$

As discussed in “Illustration of the modeling idea” section, the virtual path cost in our model is the maximum acceptable O–D cost corresponding to an O–D-based LOS requirement. Different values of *α*^*rs*^ correspond to different maximum acceptable O–D costs, which lead to different network capacity values. Also, different traffic states would lead to different maximum acceptable O–D costs, i.e., different values of *α*^*rs*^. The above property of *α*^*rs*^ offers the proposed *α*-max capacity model great flexibility to estimate the network capacity ranging from zero to physical network capacity, which will be discussed in the following Proposition 1.

#### *Remark 1*

Since $\tau _{{\min }}^{{rs}}$ only depends on the network structure, we can easily obtain *α*^*rs*^ as long as we know $\alpha ^{{rs}} \tau _{{\min }}^{{rs}}$. There are a few ways to derive $\alpha ^{{rs}} \tau _{{\min }}^{{rs}}$ in practice. First, we can conduct revealed preference or stated preference survey on travelers about their maximum acceptable cost. Second, if massive historical travel time datasets of a transportation network are available, methods developed in “Methods for determining the thresholds and representative values of *α*^*rs*^” section can be used to derive $\alpha ^{{rs}} \tau _{{\min }}^{{rs}}$. Besides, planners or managers can directly set $\alpha ^{{rs}} \tau _{{\min }}^{{rs}}$ according to their goals, and thus our model can be viewed as a tool to predict the link flows, O–D flows, and network capacity under different maximum O–D costs. These results are beneficial for planners or managers to make traffic measures, as the numerical example in “Numerical examples” section of our paper will indicate that the saturated links and the optimal O–D demand pattern may be different under different traffic states.

### Properties of the proposed model

As discussed above, the *α*-max capacity model has great flexibility to model the network capacity under various conditions due to the consideration of O–D-based LOS. This great flexibility is mathematically defined by Proposition 1.

#### **Proposition 1**

*The lower bound and the upper bound of network capacity obtained by the proposed* *α*-*max capacity model are zero and the physical capacity of transportation network, respectively.*

11 $$\phi \in \left[ {0,{\kern 1pt} {\kern 1pt} {\kern 1pt} {\kern 1pt} \phi _{{physical}} } \right]$$

#### *Proof*

In the proposed model, when *α*^*rs*^ = 1, it means that the maximum acceptable O–D cost for every O–D pair is equal to the minimum free-flow travel time. Therefore, the flow of each O–D pair should be zero in order to ensure the free-flow state, thus leading to zero as the lower bound of network capacity defined in the proposed model, i.e., $\phi _{{\min }} = 0$. Recall the excess-demand network representation in Fig. 4, if *α*^*rs*^ is a sufficiently large enough value, users are pushed to the physical routes as much as possible until the physical network reaches its physical maximum capacity. In fact, the network capacity under this condition can be derived by solving the maximum flow problem in graph theory, which has no travel choice behavior component. Consequently, the upper bound of network capacity will be the physical capacity, i.e., $\phi _{{\max }} = \phi _{{physical}}$. This completes the proof.□

#### *Remark 2*

Too small or too large value of network capacity has limited meaning for planners or managers from the practical point of view. In fact, compared with the continuous LOS, LOS categories could provide useful and important information to planners and managers. For example, typical practice is to design traffic facilities such as highway or pedestrians facilities to operate at LOS C or D (TRB 2010). Besides, planners and managers also pay more attention to network performance under some representative traffic states, such as commuting peak and off-peak. Then, two questions naturally arise: *(1) similar to the classification of highway LOS (*TRB, 2010*), can we derive thresholds of α*^*rs*^* to classify the trip LOS into different categories*? and *(2) can we get values of α*^*rs*^* corresponding to some representative traffic states*? Sect. “Methods for determining the thresholds and representative values of *α*^*rs*^” will introduce methods to obtain the thresholds and representative values of *α*^*rs*^.

#### **Proposition 2**

The *α*-max network capacity model is equivalent to two behavioral choices: one choice for deciding whether to travel or not subject to a required O–D-based LOS, and another choice for route selection according to the user equilibrium criterion.

#### Proof

First of all, we examine the Karush–Kukn–Tucker (KKT) conditions of the proposed model. As excess demands can be obtained from the travel demands via Eq. (4), we treat path flows and travel demands as explicit variables. We only attach Lagrangian multipliers *π*^*rs*^, *m*_*r*_, *n*_*s*_, and *d*_*a*_ to Eqs. (2), (5), (6) and (7), respectively, given that Eqs. (3)–(4) are the definitional constraints with respect to link flows and travel demands. Then, the Lagrangian function of the proposed model and its first-order partial derivatives with respect to path flows and travel demands can be expressed as:

12 $$\begin{gathered} L\left( {{\mathbf{f}},\;{\mathbf{q}},\;{\mathbf{\pi ,}}\;{\mathbf{d}},\;{\mathbf{m}},\;{\mathbf{n}}} \right) = Z\left( {{\mathbf{f}},\;{\mathbf{q}}} \right) + \sum\limits_{{r \in R}} {\sum\limits_{{s \in S}} {\pi ^{{rs}} \left( {q^{{rs}} - \sum\limits_{{k \in K^{{rs}} }} {f_{k}^{{rs}} } } \right)} } \hfill \\ + \sum\limits_{{r \in R}} {m_{r} \left( {\sum\limits_{{s \in S}} {q^{{rs}} } - \bar{O}^{r} } \right) + } \sum\limits_{{s \in S}} {n_{s} \left( {\sum\limits_{{r \in R}} {q^{{rs}} } - \bar{D}^{s} } \right) + \sum\limits_{{a \in A}} {d_{a} \left( {\sum\limits_{{r \in R}} {\sum\limits_{{s \in S}} {\sum\limits_{{k \in K^{{rs}} }} {f_{k}^{{rs}} \delta _{{ka}}^{{rs}} } } } - C_{a} } \right)} } \hfill \\ \end{gathered}$$

13 $$\frac{{\partial L}}{{\partial f_{k}^{{rs}} }} = \sum\limits_{{a \in A}} {t_{a} \left( {v_{a} } \right)\delta _{{ka}}^{{rs}} } - \pi ^{{rs}} + \sum\limits_{{a \in A}} {d_{a} \delta _{{ka}}^{{rs}} } = c_{k}^{{rs}} - \pi ^{{rs}} + \sum\limits_{{a \in A}} {d_{a} \delta _{{ka}}^{{rs}} }$$

14 $$\frac{{\partial L}}{{\partial q^{{rs}} }} = - u^{{rs}} {\text{ + }}\pi ^{{rs}} {\text{ + }}m_{r} {\text{ + }}n_{s}$$

where $c_{k}^{{rs}} = \sum\nolimits_{{a \in A}} {t_{a} \left( {v_{a} } \right)\delta _{{ka}}^{{rs}} }$ is the travel time of path *k* between OD pair (*r*, *s*).□

Therefore, the KKT conditions of the proposed *α*-max network capacity model with respect to path flows and travel demands are thus:

15 $$f_{k}^{{rs}} \left( {c_{k}^{{rs}} {\text{ + }}\sum\limits_{{a \in A}} {d_{a} \delta _{{ka}}^{{rs}} } - \pi ^{{rs}} } \right) = 0,\;\;\forall k \in K^{{rs}} ,\;r \in R,\;s \in S$$

16 $$c_{k}^{{rs}} {\text{ + }}\sum\limits_{{a \in A}} {d_{a} \delta _{{ka}}^{{rs}} } - \pi ^{{rs}} \ge 0,\;\;\forall k \in K^{{rs}} ,\;r \in R,\;s \in S$$

17 $$q^{{rs}} \left( {\pi ^{{rs}} + m_{r} + n_{s} - u^{{rs}} } \right) = 0,\;\;\forall r \in R,\;s \in S$$

18 $$\pi ^{{rs}} {\text{ + }}m_{r} {\text{ + }}n_{s} - u^{{rs}} \ge 0,\;\;\forall r \in R,\;s \in S$$

19 $$f_{k}^{{rs}} \ge 0,\;\;q^{{rs}} \ge 0,\;\;\forall k \in K^{{rs}} ,\;r \in R,\;s \in S$$

Equations (15)–(16) are the equilibrium conditions for the capacitated UE problem, where *d*_*a*_ is interpreted as the link delay when the link flow reaches its capacity. These two equations indicate that the travel time (including delay) on any physical path connecting an O–D pair should equal the minimum-path travel time if the flow on this path is positive. If the flow on this path is zero, its travel time must be greater than or equal to the minimum travel time. Equations (17)–(18) have a similar interpretation. If *q*^*rs*^ > 0, the generalized O–D cost (*π*_*rs*_ + *m*_*r*_ + *n*_*s*_) for users equals the maximum acceptable O–D cost (*u*_*rs*_) at the requirement of the O–D-based LOS *α*. If *q*^*rs*^ = 0, then the generalized O–D cost for users is greater than the maximum O–D cost, which is too high to induce any O–D flow into the physical network. Eq. (19) is non-negativity constraint.

According to the KKT conditions of the proposed *α*-max network capacity model, Eqs. (17)–(18) are the mechanism for deciding whether to travel or not, while Eqs. (15)–(16) are the mechanism for route choice according to the user equilibrium criterion. This completes proof.□

As can be seen from Eqs. (15)–(18), except for path travel time *c*_*k*_^*rs*^, each used physical path has an endogenous cost (i.e., $m_{r} + n_{s} + \sum\nolimits_{{a \in A}} {d_{a} \delta _{{ka}}^{{rs}} }$) which consists of origin, destination, and delay penalty costs. Actually, this endogenous cost is the maximum additional travel time that travelers are willing to pay for fulfilling the purpose of reaching the destination, which has an upper bound given by Proposition 3.

#### **Proposition 3**

*The requirement of a specified trip LOS makes each route between each O–D pair have an endogenous cost beyond the path travel time, which is upper bounded by*

20 $$m_{r} {\text{ + }}n_{s} + \sum\limits_{{a \in A}} {d_{a} \delta _{{ka}}^{{rs}} } \le \left( {\alpha ^{{rs}} - 1} \right)\tau _{{\min }}^{{rs}}$$

#### *Proof*

Given any O–D pair, the optimality conditions in Eqs. (15)–(18) indicate that all the used routes have

21 $$\left\{ \begin{gathered} c_{k}^{{rs}} {\text{ + }}\sum\limits_{{a \in A}} {d_{a} \delta _{{ka}}^{{rs}} } - \pi ^{{rs}} = 0 \hfill \\ \pi ^{{rs}} {\text{ + }}m_{r} {\text{ + }}n_{s} - u^{{rs}} = 0 \hfill \\ \end{gathered} \right.{\kern 1pt} {\kern 1pt} {\kern 1pt} {\kern 1pt} {\kern 1pt} {\kern 1pt} {\kern 1pt} {\kern 1pt} {\kern 1pt} {\kern 1pt} {\kern 1pt} {\kern 1pt} \Rightarrow {\kern 1pt} {\kern 1pt} {\kern 1pt} {\kern 1pt} {\kern 1pt} {\kern 1pt} {\kern 1pt} {\kern 1pt} {\kern 1pt} m_{r} {\text{ + }}n_{s} + \sum\limits_{{a \in A}} {d_{a} \delta _{{ka}}^{{rs}} } = u^{{rs}} - c_{k}^{{rs}}$$

Since $u^{{rs}} - c_{k}^{{rs}} \le u^{{rs}} - \tau _{{\min }}^{{rs}}$, we have $m_{r} {\text{ + }}n_{s} + \sum\limits_{{a \in A}} {d_{a} \delta _{{ka}}^{{rs}} } \le \left( {\alpha ^{{rs}} - 1} \right)\tau _{{\min }}^{{rs}}$. This completes the proof.□

Below we examine the uniqueness issue of the proposed model. Compared with the classical excess demand formulation (Beckman et al. 1956; Sheffi 1985), the three additional constraints [i.e., Eqs. (5)–(7)] are all linear, and the constant virtual path cost *u*^*rs*^ replaces the elastic demand function for each O–D pair. Thus, the proposed model is still strictly convex with respect to link flows, but linear with respect to O–D demands. This means that the uniqueness of the equilibrium links flows can be guaranteed, while the optimal O–D demands may not be unique. In order to determine a unique optimal O–D demand, Akamatsu and Miyawaki (1995) selected the solution by minimizing the closeness of the O–D matrix to some target O–D pattern. To avoid embracing any reference O–D pattern, we can add an entropy term of *q*^*rs*^ directly into the objective function of Eq. (1):

22 $$\min \;Z^{\prime}\left( {{\mathbf{f}},\;{\mathbf{q}}} \right) = \sum\limits_{{a \in A}} {\int_{0}^{{v_{a} }} {t_{a} \left( \omega \right)d\omega } } + \frac{1}{\gamma }\sum\limits_{{r \in R}} {\sum\limits_{{s \in S}} {q^{{rs}} \left( {\ln q^{{rs}} - 1} \right)} } + \sum\limits_{{r \in R}} {\sum\limits_{{s \in S}} {u^{{rs}} e^{{rs}} } }$$

where *γ* is the parameter that shall be set to a large enough value. Then, the second-order derivative of $Z^{\prime}\left( {{\mathbf{f}},\;{\mathbf{q}}} \right)$ with respect to *q*^*rs*^ is

23 $$\frac{{\partial ^{2} Z^{\prime } \left( {{\mathbf{f}},\;{\mathbf{q}}} \right)}}{{\partial q^{{rs}} \partial q^{{mn}} }} = \left\{ {\begin{array}{*{20}l} {\frac{1}{{\gamma q^{{rs}} }}} & {for\;(r,s) = (m,n)} \\ 0 & {{\text{ otherwise}}} \\ \end{array} } \right.$$

In other words, the Hessian matrix in terms of *q*^*rs*^ is positive definite, which means that the objective function is strictly convex with respect to *q*^*rs*^, and thus the optimal O–D demands can be determined uniquely. We should point out that the entropy term in Eq. (22) is added just for ensuring a unique solution of the optimal O–D demand, which is different from the entropy term in the combined trip distribution-assignment model. Besides, we can also use stochastic user equilibrium to replace the UE for obtaining a unique solution of the optimal O–D demand, which is however not the main focus of this paper.

### Methods for determining the thresholds and representative values of *α*^*rs*^

As the uncertainties of traffic supply and demand exist in the transportation network, traffic states between an O–D pair will not be unchanged, leading to travel time variability. One can aggregate massive data of individual trips collected by sensors (e.g., floating cars) during a long-term period into travel time datasets (*TT*_*t*_^*rs*^) by O–D pair (*r*, *s*) and time period *t*:

24 $${\text{TT}}_{{\text{t}}}^{{rs}} = \left\{ {i|o(i) \in z(r),d(i) \in z(s),\left\lfloor {\vartheta (i)} \right\rfloor = t} \right\}$$

where *o*(*i*) and *d*(*i*) are the starting and ending points of trip *i*, respectively; *z*(*r*) is the geographic region of zone *r*, $\left\lfloor {\vartheta (i)} \right\rfloor$ is the rounded start time of trip *i* using the floor operator $\left\lfloor \cdot \right\rfloor$. For simplicity, let $\tau _{{\min }}^{{rs}} ,{\kern 1pt} {\kern 1pt} {\kern 1pt} {\kern 1pt} {\kern 1pt} \tau _{\rho }^{{rs}} ,{\kern 1pt} {\kern 1pt} {\kern 1pt} {\kern 1pt} \text{and} {\kern 1pt} {\kern 1pt} {\kern 1pt} {\kern 1pt} \tau _{{\max }}^{{rs}}$ denote the minimum travel time, *ρ* percentile of travel time and maximum travel time in travel time datasets.

**Fig. 6:**
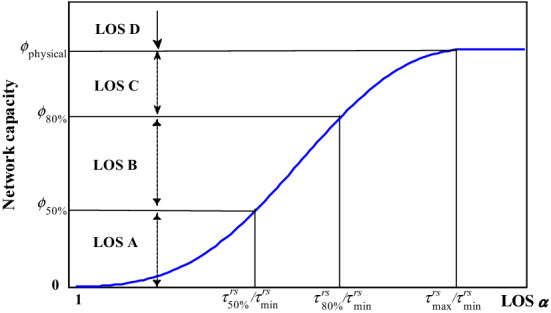
The illustration of O–D based LOS classes and network capacities

First of all, we provide a general framework based on on-time arrival probability to determine thresholds for classifying the trip LOS. The maximum acceptable O–D cost is the price that a traveler is willing to pay for travel, which actually depends on his/her requirement of on-time arrival probability. If the traveler requires a *ρ* on-time arrival probability (e.g., 80%), his/her maximum acceptable O–D cost must be greater than or equal to $\tau _{\rho }^{{rs}}$, and the corresponding *α*^*rs*^ is $\tau _{\rho }^{{rs}} /\tau _{{\min }}^{{rs}}$. Obviously, the on-time arrival probability of $\tau _{{\min }}^{{rs}}$ and ${\kern 1pt} {\kern 1pt} \tau _{{\max }}^{{rs}}$ are 0 and 100%, respectively. If we choose 50%, 80% as another two requirements of on-time arrival probability, we will have four thresholds of *α*^*rs*^ (i.e., 1, $\tau _{{50\% }}^{{rs}} /\tau _{{\min }}^{{rs}}$,$\tau _{{80\% }}^{{rs}} /\tau _{{\min }}^{{rs}}$, and $\tau _{{\max }}^{{rs}} /\tau _{{\min }}^{{rs}}$) to classify the trip LOS and its corresponding capacity into four LOS classes from A to D as shown in Fig. 6. Compared with the highway LOS categories defined in the Highway Capacity Manual (HCM) 2010 (TRB 2010), we can have a similar interpretation of these O–D-based LOS classes. For example, when $\alpha ^{{rs}} \in \left( {\tau _{{80\% }}^{{rs}} /\tau _{{\min }}^{{rs}} ,{\kern 1pt} {\kern 1pt} {\kern 1pt} {\kern 1pt} \tau _{{\max }}^{{rs}} /\tau _{{\min }}^{{rs}} } \right]$, the transportation network can provide LOS C of network capacity, and the maximum network capacity under LOS C is the network capacity corresponding to $\alpha ^{{rs}} = \tau _{{\max }}^{{rs}} /\tau _{{\min }}^{{rs}}$. In fact, this network capacity is the maximum physical amount of flow that the network can accommodate, which is similar to the highway operational capacity defined by LOS E in HCM 2010.

Although the thresholds are useful in obtaining the range of network capacity under specified LOS class, the network capacities under some representative traffic states such as morning peak are also key concerns for planners and managers. Then, we can further use machine learning methods to obtain the values of *α*^*rs*^ under representative states for reference to planners and managers. Since *α*^*rs*^ is dimensionless, the travel time datasets of all trips *TT*_*t*_^*rs*^ are normalized by the minimum free-flow travel time *FFTT*^*rs*^ of O–D pair (*r*, *s*):

25 $$NTT_{{\text{t}}}^{{rs}} = \frac{{TT_{{\text{t}}}^{{rs}} }}{{{\text{FFTT}}^{{rs}} }}$$

Note that *NTT*_*t*_^*rs*^ corresponds to *α*^*rs*^ at time period *t*. Machine learning methods such as K-means clustering algorithm can be used to cluster *NTT*_*t*_^*rs*^ into *n* classes (*n* is the number of classes that we want to classify the traffic states of transportation network). The values of clustering center points of *n* classes are the representative values of *α*^*rs*^, i.e., *α*_1_^*rs*^, *α*_2_^*rs*^, …, and *α*_*n*_^*rs*^ corresponding to *n* classes of the traffic states. Besides the K-means algorithm, other valid clustering algorithms are also applicable. For example, Cheng et al. (2020) used the improved fuzzy c-means clustering approach to classify the urban traffic states.

It should be noted that herein we just provide a general framework and machine learning methods for deriving thresholds of *α*^*rs*^ to classify the trip LOS and obtaining the values of *α*^*rs*^ under representative states. In practice, the numbers of LOS classes and the associated thresholds, and number of classes of traffic states should be customized judiciously according to local traffic states and planners’ requirements. For instance, the LOS F threshold in HCM 2010 for analyzing freeway weaving segments has changed compared to that in HCM 2000 (TRB 2000). Besides, by using K-means clustering algorithm, Yan (2019) identified three representative values *α*_1_^*rs*^ = 1.124, *α*_2_^*rs*^ = 1.807, and *α*_3_^*rs*^ = 2.384 to study the ridesharing problem of the urban transportation network of Shenzhen, China. These three values were interpreted as representations of the off-peak travel time in the night time, the travel time of the transition period from off-peak to peak, and the peak travel time, respectively.

## Solution Algorithm

Due to the same structure with the excess demand formulation, Akamatsu and Miyawaki (1995) adopted the Frank-Wolfe algorithm to solve their model. In comparison with the excess demand formulation, the proposed *α*-max capacity model has three additional side constraints [i.e., Eqs. (5)–(7)]. As a result**,** the Cartesian product structure of the feasible set as in the conventional excess demand model is destroyed, rendering a more computationally demanding model (Patriksson 2004, 2015; Nie et al. 2004). Hence, solving the *α*-max capacity model is much more complex than the conventional excess demand model without side constraints. Converting the TAP with side constraints into a sequence of TAP subproblems through the use of penalties or Lagrangian multipliers is a popular method to solve the TAP with side constraints. For example, the Lagrangian multiplier method (Larsson and Patriksson 1995; Nie et al. 2004) and the penalty function method (Inouye 1987; Yang and Yagar 1994, 1995; Nie et al. 2004) have been developed for solving the capacitated TAP. However, the above methods are sensitive to factors like the solution accuracy of subproblems and the penalty parameter sequence (Nie et al. 2004), and thus have difficulty to converge if inappropriate penalty values are used (Ryu et al. 2014b).

Although there are some existing algorithms that aim to resolve solution accuracy of subproblems, e.g., origin-based algorithm (Shi et al. 2015) and path-based greedy algorithm (Feng et al. 2020), we use a soft constraint technique to handle the three additional constraints of the proposed model for two reasons. Firstly, the soft constraint technique always guarantees the feasible solution because the penalization of violating side constraints is imposed in the objective function instead of destroying the Cartesian product structure of the feasible set. Secondly, with the penalized terms in the objective function, the three additional constraints of the proposed model can be transformed into Beckmann-like terms, and the proposed model is thus transformed into a generalized elastic demand UE traffic assignment model. This means that we can use the existing widely used algorithms to solve the proposed *α*-max capacity model.

Specifically, the proposed model is rewritten as:

26 $$\begin{gathered} \min \;Z\left( {{\mathbf{f}},\;{\mathbf{q}}} \right) = \sum\limits_{{a \in A}} {\int_{0}^{{v_{a} }} {t_{a} \left( \omega \right)d\omega } } + \sum\limits_{{a \in A}} {\int_{0}^{{v_{a} }} {p_{a} \left( \omega \right)d\omega } } + \sum\limits_{{r \in R}} {\sum\limits_{{s \in S}} {u^{{rs}} e^{{rs}} } } \\ + \sum\limits_{{r \in R}} {\int_{0}^{{O^{r} }} {p_{r} \left( \omega \right)d\omega } } + \sum\limits_{{s \in S}} {\int_{0}^{{D^{s} }} {p_{s} \left( \omega \right)d\omega } } \\ \end{gathered}$$

subject to Eqs. (2)–(4) and (8), where *O*_*r*_ and *D*_*s*_ are the trip production at origin *r* and the trip attraction at destination *s*; and *p*_*r*_(·), *p*_*s*_(·), and *p*_*a*_(·) are the soft penalty functions for relaxing Eqs. (5)–(7), respectively.

The following exponential-form soft penalty function has been shown to successfully relax the hard capacity constraint in solving the capacitated TAP (Nguyen et al. 2001; Noh 2013; Ryu et al. 2017):

27 $$p_{a} = \frac{{x_{a} }}{{C_{a} }} \cdot \exp \left( {\theta \cdot \left( {x_{a} - C_{a} } \right)} \right)$$

where *θ* is a parameter. We use the above exponential-form soft penalty function to relax the other two side constraints in Eqs. (5)–(6) of our proposed model.

28 $$\begin{gathered} p_{r} = \frac{{O^{r} }}{{\overline{O} ^{r} }} \cdot \exp \left( {\theta \cdot \left( {O^{r} - \overline{O} ^{r} } \right)} \right){\kern 1pt} \hfill \\ p_{s} = \frac{{D^{s} }}{{\overline{D} ^{s} }} \cdot \exp \left( {\theta \cdot \left( {D^{s} - \overline{D} ^{s} } \right)} \right) \hfill \\ \end{gathered}$$

In fact, *p*_*a*_, *p*_*r*_ and *p*_*s*_ in soft constraint technique are used to approximate the Lagrangian multipliers *d*_*a*_, *m*_*r*_, and *n*_*s*_ associated with Eqs. (7), (5) and (6). In other words, the path cost and O–D cost in the elastic demand UE traffic assignment model are respectively replaced by:

29 $$gc_{k}^{{rs}} = \sum\limits_{{a \in A}} {\left( {t_{a} \left( {v_{a} } \right) + p_{a} \left( {v_{a} } \right)} \right)\delta _{{ka}}^{{rs}} } ,\;\;\forall k \in K^{{rs}} ,\;r \in R,\;s \in S$$

30 $$g\pi ^{{rs}} = gc_{{\bar{k}^{{rs}} }}^{{rs}} + p_{r} + p_{s} ,\;\;\forall r \in R,\;s \in S$$

where $\bar{k}^{{rs}}$ is the shortest path of O–D pair (*r*, *s*).

**Fig. 7:**
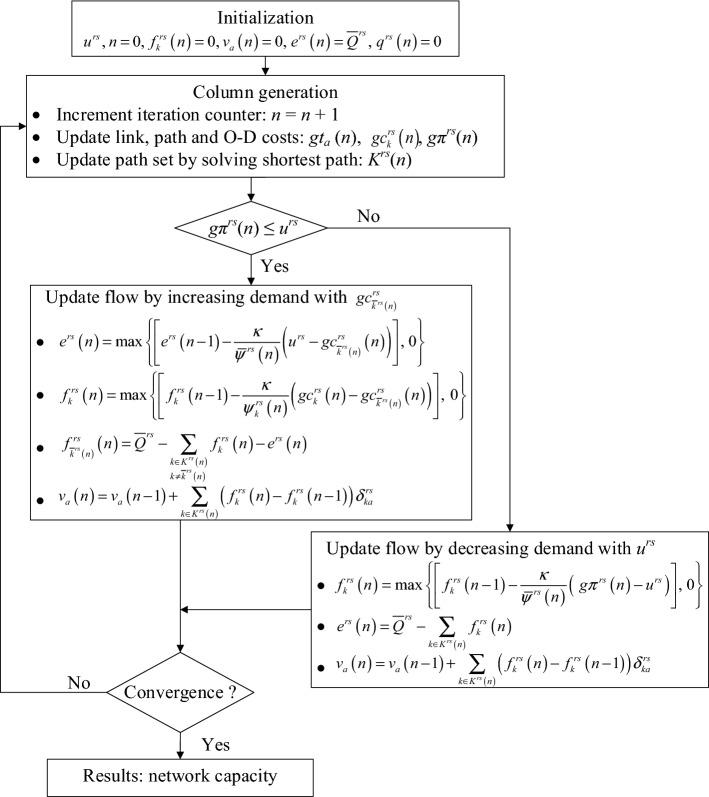
Flowchart of the modified gradient projection algorithm with soft constraint technique for solving the proposed model


Therefore, the gradient projection algorithm developed by Ryu et al. (2014a) for solving the elastic demand TAP can be adopted to solve our proposed model along with the above soft constraint technique. In the modified gradient projection algorithm with soft constraint technique, the flow update per iteration is conducted as follows: (1) if the calculated O–D cost in iteration *n* is smaller than its corresponding virtual path cost, it means the O–D actual congestion level is lower than the maximum acceptable level and thus more O–D flows should be pushed into the physical network; and (2) if the O–D actual congestion level is higher than the maximum acceptable level, more O–D flows should be pushed into the virtual path. Three modifications are also made to the gradient projection algorithm developed by Ryu et al. (2014a): (1) the second-order derivative of the objective function in Eq. (26) with respect to the virtual path flow is zero because *u*^*rs*^ is a constant number rather than an excess demand function *W*^*rs*^(^.^) as in Ryu et al. (2014a); (2) a new stopping criterion, i.e., the relative gap (RG) $\text{RG} = \frac{{\sum\limits_{{r \in R}} {\sum\limits_{{s \in S}} {\sum\limits_{{k \in K^{{rs}} }} {f_{k}^{{rs}} (n)\left( {gc_{k}^{{rs}} (n) - gc_{{\overline{k} ^{{rs}} \left( n \right)}}^{{rs}} (n)} \right)} } } }}{{\sum\limits_{{r \in R}} {\sum\limits_{{s \in S}} {\sum\limits_{{k \in K^{{rs}} }} {gc_{k}^{{rs}} (n)f_{k}^{{rs}} (n)} } } }} \le \varepsilon$, is used where *n* is the number of the increment iteration of the algorithm; and (3) note that column generation is used to generate the shortest paths based on the current link travel times and augment the path set with new paths, so in the first iteration there is only one shortest path for each O–D pair and *RG* will be equal to zero. The overall flowchart of the modified gradient projection algorithm with soft constraint technique is shown in Fig. 7, and its detailed solution procedure is as follows:

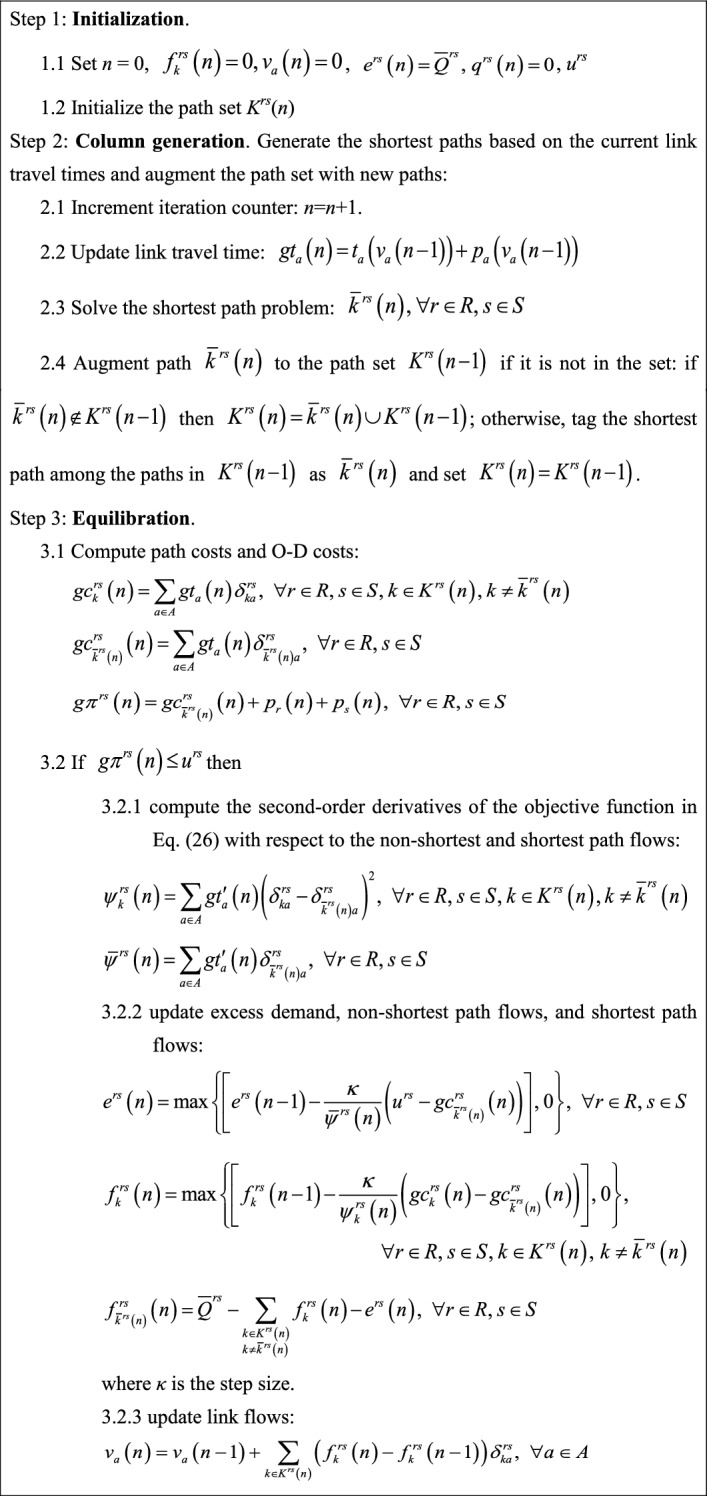



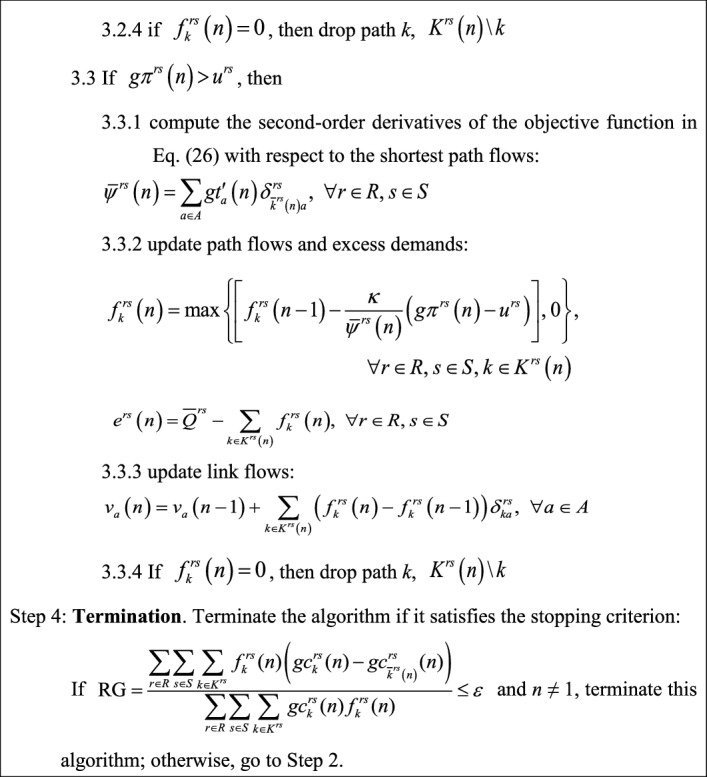


## Numerical examples

In this section, two networks are used to illustrate the features and applicability of the proposed model. For the parameters used in the models, we use a unified *α* for all O–D pairs for simplicity, although the parameter *α*^*rs*^ should be O–D pair specific. The maximum potential travel demands $\bar{Q}^{{rs}}$ are assumed to be 2 times of the current O–D demands, and the maximum trip production $\bar{O}^{r}$ and attraction $\bar{D}^{s}$ are assumed to be 1.8 times of the current trip production and attraction. For the parameters used in the solution algorithm, the parameter *θ* in the soft penalty function is set as 1. The step size *κ* is 0.4. The tolerance error for the stopping criterion (RG) is set at 1E-6. The solution algorithm is coded in Intel Visual FORTRAN XE, and run on a 3.40 GHz processor with 16.00 GB of RAM.

### Example 1: grid network

**Fig. 8:**
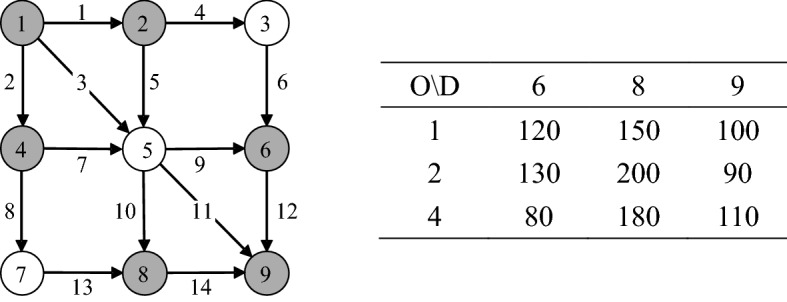
The grid network and its current O–D trip table (veh/min)

The grid network as depicted in Fig. 8 consists of 9 nodes, 14 directed links, and 9 O–D pairs. Nodes 1, 2, and 4 are origin nodes; while nodes 6, 8, and 9 are destination nodes (all shaded). The current O–D trip table is also given in Fig. 8. Table 2 gives the characteristics of 14 links. We use the standard Bureau of Public Road function as the link travel time function.

**Table 2:** Link characteristics of the grid network

Link	Node		Capacity (veh/min)	Free-flow travel time (min)
	From	To		
1	1	2	280	2
2	1	4	290	1.5
3	1	5	280	3
4	2	3	280	1
5	2	5	600	1
6	3	6	300	2
7	4	5	500	2
8	4	7	400	1
9	5	6	500	1.5
10	5	8	700	1
11	5	9	250	2
12	6	9	300	1
13	7	8	350	1
14	8	9	220	1

In the following analysis, we will explore the impact of O–D-based LOS on the network capacity, provide examples to illustrate how our model supports traffic management schemes, and test the validity of the soft constraint technique in handling hard constraints.

1. * The Impact of O–D-based LOS on the Resultant Network Capacity*

**Fig. 9:**
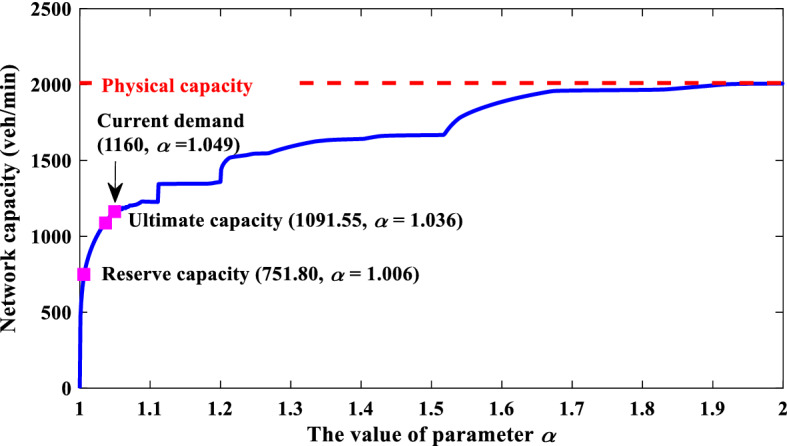
Reserve capacity, ultimate capacity, current demand, and the relationship between network capacity and the value of parameter *α* for the grid network.

Below we examine the impact of different requirements of O–D-based LOS (i.e., different values of parameter *α*) on the resultant maximum capacity obtained from our model. For comparison purposes, the reserve capacity (Wong and Yang 1997) and ultimate capacity (Yang et al. 2000; Chen and Kasikitwiwat 2011; Chen et al. 2013) are also calculated. The impedance parameter for trip distribution in the ultimate capacity model is 0.75. As can be seen from Fig. 9, the reserve capacity is 751.80 (veh/min) and its corresponding *α* equals 1.006, while the ultimate capacity is 1091.55 (veh/min) and its corresponding *α* equals 1.036. Both the reserve capacity and ultimate capacity are lower than the current demand (i.e., the summation of current travel demands). In addition, there is only 1 saturated link but 4 zero-flow links, and the average V/C ratio of all 14 links is only 0.34 for the reserve capacity, while there are 3 saturated links but 4 zero-flow links, and the average V/C ratio of all 14 links is only 0.46 for the ultimate capacity. Therefore, the reserve capacity and ultimate capacity may underestimate the real network capacity. On the contrary, as shown in Fig. 9, the network capacity from our model firstly increases rapidly and then gradually stabilizes with the increase of *α*. This means that the marginal benefit of the increase of network capacity through the increase of the maximum acceptable O–D cost decreases and finally disappears until the network capacity reaches its physical capacity.

Hence, the *α*-max capacity model provides great flexibility to model the network capacity from zero to physical capacity. Due to this flexibility, the existing reserve capacity and ultimate capacity models can be viewed as special cases of our proposed model as shown in Fig. 9, despite that their corresponding LOS requirements are not known a prior. As discussed in “Methods for determining the thresholds and representative values of *α*^*rs*^” section, we can also classify the continuous trip LOS *α* in Fig. 9 into different discrete LOS categories through thresholds of *α*. For example, the values of *α* corresponding to the reserve capacity, the current demand, and the physical capacity can be used as thresholds to obtain four discrete LOS categories from LOS A to LOS D. Then, similar to the highway operational capacity defined by LOS E in HCM (TRB 2010), the maximum network capacity under LOS C is the maximum physical amount of flow that the grid network can accommodate.

(2) *The Support of Our Model for Traffic Management Schemes*

It is also interesting to find that with the increase of the value of *α*, it is not necessary that all links always become more saturated as shown in Table 3. For example, link 2 is saturated in the ultimate capacity but not saturated when *α* = 1.5 and 2; link 14 is saturated when *α* = 1.5 but not saturated when *α* = 2. Note that the link capacity is a main barrier to prevent the increase of network capacity. This phenomenon implies that the critical degrees of links under different LOS requirements can be different. In other words, different links may become the active constraints under different network capacities due to the network effect. It is therefore not appropriate to conduct capacity enhancement decisions only according to the traffic states under the current travel demand and traffic operation conditions. In contrast, the proposed *α*-max capacity model enables to identify different critical or saturated links under different traffic states, which is important for planners and managers to implement flexible management measures such as capacity enhancement for the specific links rather than fixed links.

**Table 3:** Saturated links under different values of *α*

Saturated links\capacity	Reserve	Ultimate	*α* = 1.5	*α* = 2
Number	1	3	5	7
Link no.	13	**2**, 5, 13	3, 5, 11, 13, **14**	3, 4, 5, 7, 10, 11, 13

Except for identifying different critical links under traffic states, the *α*-max capacity model can also support choosing a better road pricing scheme. Suppose that *α* = 1.5 for the grid network and traffic managers need to lower the network capacity through road pricing on link 3, link 5, and link 7. The function of the toll charge is given by:

31 $$t_{a} \left( {v_{a} ,\;x_{a} } \right) = \left\{ {\begin{array}{*{20}l} {\left( {1 + x_{a} /x_{a} \beta _{{VOT}} - \beta _{{VOT}} } \right),} & {a \in \bar{A}} \\ {t_{a} \left( {v_{a} } \right)t_{a} \left( {v_{a} } \right),} & {a \in A\backslash \bar{A}} \\ \end{array} } \right.$$

where *x*_*a*_ is the toll charge, *β*_VOT_ is the value of time, and $\bar{A}$ is the set of charged links, i.e., link 3, link 5, and link 7. Then our model can be used as a tool to assess the network capacity under different toll charge levels and Table 4 gives resultant network capacities and O–D flows.

**Table 4:** The resultant network capacities and O–D flows under different levels of toll charge for *α* = 1.5

Toll charge (%)	O–D (1, 6)	O–D (1, 8)	O–D (1, 9)	O–D (2, 6)	O–D (2, 8)	O–D (2, 9)	O–D (4, 6)	O–D (4, 8)	O–D (4, 9)	Network capacity
*x*_*a*_/*β*_VOT_ = 0	240	226	200	194	382	180	160	0	90	1672
*x*_*a*_/*β*_VOT_ = 10	240	226	200	194	382	180	160	0	89	1671
*x*_*a*_/*β*_VOT_ = 20	240	226	200	194	377	180	160	0	70	1647
*x*_*a*_/*β*_VOT_ = 30	240	226	200	194	323	180	160	0	61	1584
*x*_*a*_/*β*_VOT_ = 40	240	226	200	194	245	180	160	0	60	1505
*x*_*a*_/*β*_VOT_ = 50	240	130	200	194	226	180	160	0	60	1390
*x*_*a*_/*β*_VOT_ = 60	240	130	200	194	226	180	160	0	60	1390
*x*_*a*_/*β*_VOT_ = 70	240	130	200	194	226	180	160	0	60	1390
***x***_***a***_/***β***_**VOT**_** = 80**	240	130	200	260	117	180	**94**	0	60	**1281**
*x*_*a*_/*β*_VOT_ = 90	240	130	200	260	22	180	0	0	60	1092
*x*_*a*_/*β*_VOT_ = 100	240	130	200	260	0	180	0	0	60	1070
*x*_*a*_/*β*_VOT_ = 110	240	130	200	260	0	180	0	0	60	1070
*x*_*a*_/*β*_VOT_ = 120	240	130	200	191	0	180	0	0	60	1001
*x*_*a*_/*β*_VOT_ = 130	240	129	196	40	0	180	0	1	59	845
*x*_*a*_/*β*_VOT_ = 140	240	127	191	40	0	161	0	3	57	818
*x*_*a*_/*β*_VOT_ = 150	240	124	194	40	0	0	0	6	54	658

As can be seen from Table 4, with the increase of the toll charge, the network capacity and the flow of O–D pairs change quite differently. Specifically, the network capacity generally decreases. The flow of O–D pair (1, 6) keeps unchanged; while flows of O–D pairs (1, 8), (2, 8), (4, 6), and (4, 9) decrease at different levels; but the flow of O–D pair (2, 6) first increases and then decreases. The detailed change tendency of O–D flows, link flows and network capacity enables traffic managers to determine a reasonable toll charge according to their realistic needs. For example, if they need to lower network capacity by at least 20% while satisfying at least 50% of the travel demand of the O–D pair (4, 6), then *x*_*a*_/*β*_VOT_ = 80% would be a good choice for traffic managers as the network capacity has been decreased by 23.39% (i.e., (1672–1281)/1672 = 23.39%) while satisfying 58.75% (i.e., 94/160 = 58.75%) of travel demand of O–D pair (4, 6).

(3) *The validity of the Soft Constraint Technique in Handling Hard Constraints.*

Last, we examine the effectiveness of the soft constraint technique in handling the three hard constraints. The proposed model with hard constraints can be treated as the benchmark but the three hard constraints make it quite difficult to solve; the proposed model with soft constraints can be treated as an approximation model but with significant computational tractability due to the same structure with the classical elastic demand UE model (Sheffi 1985). Table 5 presents the assigned link flows and the maximum O–D demands obtained from the proposed models with hard constraints and soft constraints, respectively. One can see that the results from the proposed models with hard constraints and soft constraints are quite close with the maximum error of 1.95% for link volumes and 3.04% for maximum O–D demands. Therefore, the soft constraint technique can have a high-quality accuracy in handling hard constraints while ensuring the existence of a feasible solution and computational efficiency. Next section will further test the efficiency of the soft constraint technique in handling the three hard constraints for large-scale network application.

**Table 5:** The results of link volumes and maximum O–D demands from the proposed models with hard constraints and soft constraints (*α* = 1.5)

Link Volume (veh/min)								Maximum O–D Demand (veh/min)			
Link	Hard	Soft	Error	Link	Hard	Soft	Error	O–D	Hard	Soft	Error
1	99.60	101.27	1.67%	**10**	478.00	476.42	0.33%	**(1, 6)**	240	240.00	0.00%
2	286.40	285.29	0.39%	**11**	250.00	248.41	0.64%	**(1, 8)**	226	225.29	0.31%
3	280.00	278.74	0.45%	**12**	0.00	0.00	NA	**(1, 9)**	200	200.00	0.00%
4	255.60	256.43	0.33%	**13**	350.00	349.78	0.06%	**(2, 6)**	194	192.56	0.74%
5	600.00	599.44	0.09%	**14**	220.00	218.85	0.52%	**(2, 8)**	382	382.05	0.01%
6	255.60	256.43	0.33%					**(2, 9)**	180	180.00	0.00%
7	186.40	182.77	**1.95%**					**(4, 6)**	160	160.00	0.00%
8	350.00	349.78	0.06%					**(4, 8)**	0	0	NA
9	338.40	336.13	0.67%					**(4, 9)**	90	87.26	**3.04%**

Error =|hard-soft|/hard × 100%

### Example 2: winnipeg network

**Fig. 10:**
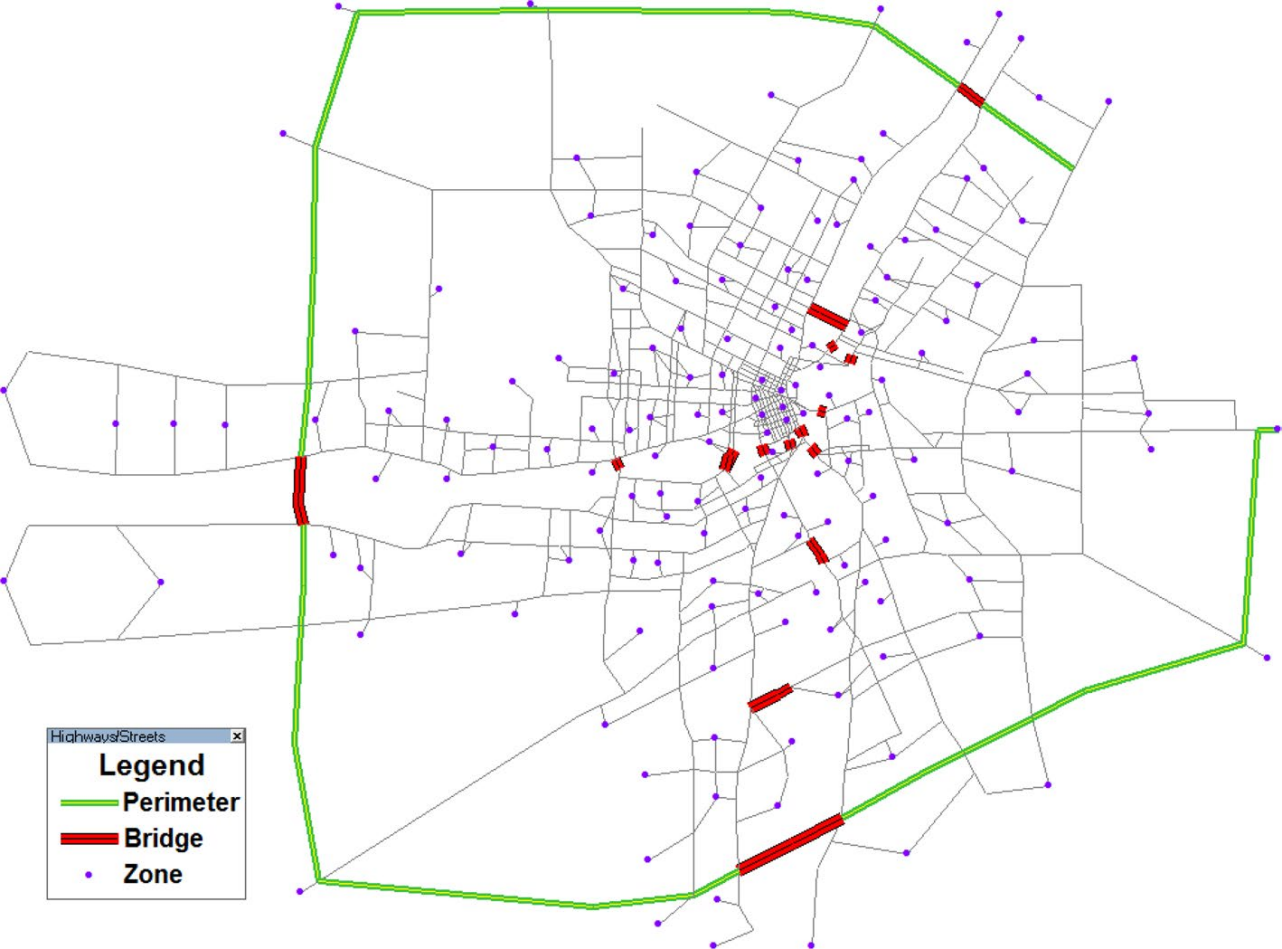
Winnipeg network

In this section, we use the Winnipeg network as shown in Fig. 10 in Manitoba, Canada to demonstrate the applicability of the *α*-max capacity model and the solution algorithm in large-scale networks. The Winnipeg network consists of 154 zones, 1067 nodes, 2535 links, and 4,345 O–D pairs. The network structure, O–D trip table, and link performance parameters are obtained from the Emme/4 software (INRO Consultants 1999). Recall that it is quite challenging to develop an efficient algorithm for solving BLP in large networks. Therefore, in this example we only solve the reserve capacity BLP model through the incremental traffic assignment method for comparsion. When the reserve capacity multiplier *μ* is equal to 0.548, all links can satisfy the link capacity constraints and the reserve capacity is 29844 (veh/h).

**Fig. 11:**
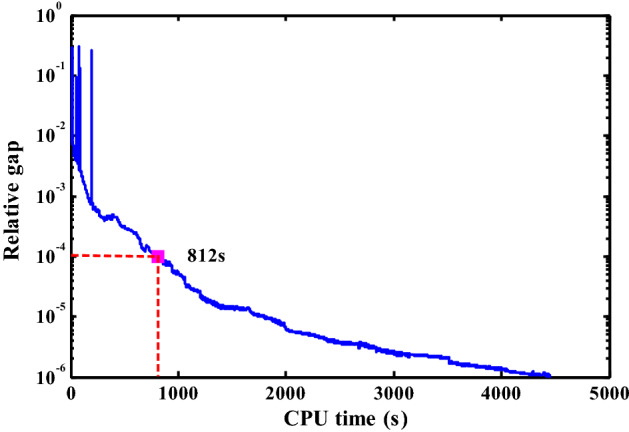
The convergence curve of the solution algorithm (*α* = 1.5)

**Fig. 12:**
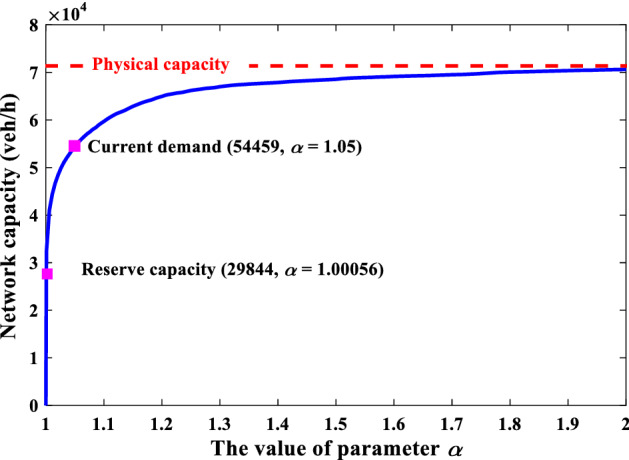
The network capacity as a function of *α* in the Winnipeg network

Without loss of generality, Fig. 11 depicts the convergence curve of the solution algorithm at *α* = 1.5. The CPU time for *α* = 1.5 is 4454 s. Note that the tolerance error of the relative gap is set at 1E-6, which is much stricter than the typical value of 1E-4 suggested by Boyce et al. (2004). If the tolerance error is set at 1E-4, the CPU time for *α* = 1.5 will be 812 s, i.e., 18.24% of 4454 s. The above results verify the efficiency of the solution algorithm in solving the proposed model in large-scale networks. Then, we examine the effect of the O–D-based LOS requirement (i.e., different values of *α*) on the resultant maximum capacity in the Winnipeg network as shown in Fig. 12. Consistent with the grid network, the proposed *α*-max capacity offers great flexibility to model the Winnipeg network capacity in a broad range from 0 to 72,531 (veh/h) (i.e., physical capacity), including the reserve capacity (29,844 veh/h) and the current demand (54,459 veh/h) as special cases of our proposed model despite that their corresponding LOS requirements are not known *a prior*. In fact, the corresponding values of *α* for the reserve capacity and current demand are 1.00056 and 1.050, respectively. There is a large gap between the reserve capacity and the current demand (24,615 veh/h) as well as the gap between the reserve capacity and the physical capacity (42,687 veh/h). Besides, in the reserve capacity model, only one link reaches its capacity constraint and the average V/C ratio of all links is only 0.19. If we run the UE assignment with the current O–D trip table in this network, 195 links (i.e., 10.14% of all the 1923 physical links excluding the centroid connectors) have flows exceeding their capacities (i.e., *v*_*a*_ > *C*_*a*_). The above deviation (i.e., only one saturated link versus about 10% saturated links) indicates the reserve capacity model may underestimate the network capacity and thus is unreasonable for a real transportation network. Similarly, a highly overestimated network capacity such as the physical capacity without behavioral consideration also provides limited information to transportation planners concerning the network performance (Yang et al. 2000).

Therefore, it is necessary to classify the continuous trip LOS *α* into different discrete LOS categories and identify different maximum network capacities under different trip LOS categories. The methods presented in “Methods for determining the thresholds and representative values of *α*^*rs*^” section could support such a classification. Similar to the gird network, the values of *α* corresponding to the reserve capacity, the current capacity, and the physical capacity can be used as thresholds to obtain four discrete LOS categories from LOS A to LOS D. Then, the maximum network capacity under LOS C is the maximum physical amount of flow that the Winnipeg network can accommodate, which corresponds to the definition of the highway operational capacity defined by LOS E in HCM (TRB 2010).

**Fig. 13:**
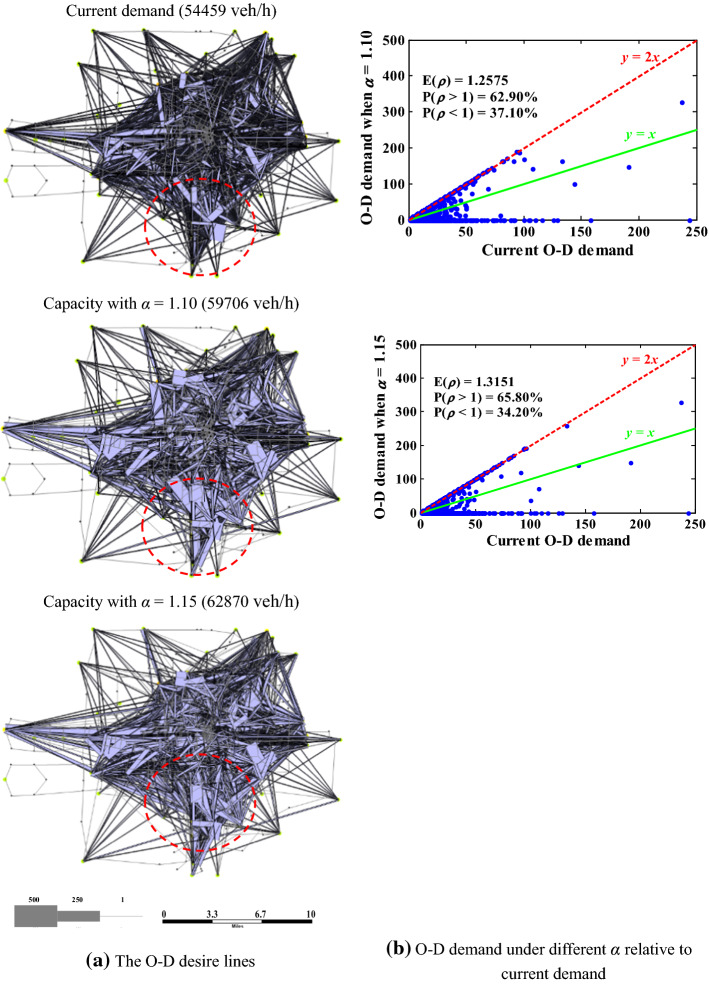
Maximum O–D demand patterns of different network capacities (*ρ*: the ratio of the maximum O–D demand obtained from the proposed model to the current demand)

Finally, we examine the maximum O–D demand pattern under different network capacity states. Note that the reserve capacity is a uniform scaling of the current O–D demand. Figure 13a plot the desire lines of the network capacities with *α* = 1.10 and *α* = 1.15 and the current demand, and Fig. 13b quantifies the difference relative to the current O–D demand. One can see that: (1) the network capacity is related to the O–D demand pattern rather than a pure supply-side indictor. Relative to the current O–D demand, the network capacity still increases by 9.63% (or 15.44%) despite that 37.10% (or 34.20%) of O–D pairs have decreased demands for *α* = 1.10 (or *α* = 1.15) as shown in Fig. 13b; and (2) it is not necessary that all O–D demands always change proportionally with the increase of network capacity as shown in Fig. 13a. In Fig. 13a, with the increase of network capacity, the width of various desire lines does not have the same change tendency as marked by the red dashed circles. This means that under different traffic states, different optimal O–D demand patterns match the network structure. The identified different optimal/maximum O–D demand patterns under various traffic states can influence the development of customized demand management strategies to adjust the O–D demand structure. This further verifies that the proposed model can support traffic managers to adopt flexible management and control measures according to traffic states.

## Concluding remarks

In this paper, we proposed the *α*-max network capacity model with the O–D-based LOS requirement to surmount the disadvantages associated with the existing MP-based network capacity models, i.e., the inconsideration of LOS in single-level MP and non-convexity of BLP. Mathematically, the *α*-max network capacity model was formulated as a single-level MP using the excess-demand network augmentation. Specifically, the virtual path cost for each O–D pair in the augmented network was determined by trip LOS requirement, which makes the proposed model flexible to estimate the network capacity under various traffic conditions. For practical use, we further provided methods to convert continuous trip LOS into discrete trip LOS categories and to obtain the network capacity under representative traffic states. This classification is useful in supporting the development of active management measures, such as setting the maximum trip cost as the desired value to control the network capacity for special purposes. A modified gradient projection algorithm with soft constraint technique was developed to solve the proposed method. Two networks were used to examine the features of the proposed model and the efficiency of the solution algorithm.

In summary, the *computational and behavioral advantages* of the proposed *α*-max capacity model compared to the existing network capacity models are as follows:

- It consider the trip LOS requirement, which offers flexibility to estimate the network capacity from zero to physical capacity. The existing reserve capacity and ultimate capacity models can be viewed as special cases of the proposed model despite that their corresponding LOS requirements are not known *a prior*. This flexibility also makes it possible to identify different critical or saturated links according to different traffic states, which is useful to support meticulous management and control.
- The consideration of O–D-based (i.e., trip) LOS has two benefits compared with the link-based LOS. Firstly, the O–D-based LOS requirement can intuitively reflect the maximum travel cost, which is the price that travelers are willing to pay for a travel and thus more important than the operating conditions of a single link (i.e., the link-based LOS) for travelers. Secondly, planners and managers usually pay more attention to the O–D travel time and even directly set certain O–D travel time as one of their planning targets. Consequently, the consideration of O–D-based LOS in the proposed model can help planners and managers know how many traffic flows the transportation network can accommodate under a certain requirement of O–D-based LOS. The result is a key information for planners and managers to take flexible and effective measures for guaranteeing their planning targets.
- It is convex and single-level MP, which is convenient for developing a computationally efficient solution algorithm for large-scale network applications. This has been verified in the Winnipeg network.

For future research, several directions are worthy of further investigations. It is interesting to integrate the concept of reserve capacity into the proposed model. In other words, we can use the current O–D cost to replace the shortest free-flow path cost *τ*_min_ in the virtual path cost for each O–D pair, which means that we preserve the fixed O–D cost pattern just like the fixed O–D demand pattern preserved in the reserve capacity model. Also, we plan to extend the proposed model to a multi-modal transportation network with both physical capacity constraints and environmental constraints (Chen et al. 2011b). Besides, we can explore various applications of the proposed model, such as car ownership estimation, network design, environmental capacity estimation, etc. At last, since the MP-based approach and the MFD approach are two different approaches for estimating the network capacity, it is interesting and important to collect consistent data for these two approaches and then investigate their relationship (particularly whether there are analytical linkages) in future studies.

## Data Availability

Open dataset available at https://github.com/bstabler/TransportationNetworks.

## List of symbols

*a*, *A*: Link and the set of directed links in the network
*u*: Virtual path travel time
*α*: Level-of-service requirement
*τ*_min_: The free-flow travel time of the shortest path between a O–D pair
*r*, *s*: Origin and destination
*v*_*a*_, *t*_*a*_, *C*_*a*_: Traffic flow, travel time and capacity of link *a*
*q*^*rs*^, *e*^*rs*^, $\bar{Q}^{{rs}}$: Realized demand, excess demand and maximum potential demand between O–D pair (*r*, *s*)
*f*_*k*_^*rs*^: The flow on path *k* between O–D pair (*r*, *s*)
*K*^*rs*^: The set of paths connecting O–D pair (*r*, *s*)
$\bar{k}^{{rs}}$: The shortest path of O–D pair (*r*, *s*).
$\delta _{{ka}}^{{rs}}$: Link-path incidence indicator
$\bar{O}^{r}$, $\bar{D}^{s}$: The maximum trip production at origin *r* and the maximum trip attraction at destination *s*
$$\phi$$: Network capacity
*π*^*rs*^, *m*_*r*_, *n*_*s*_, *d*_*a*_: Lagrangian multipliers
$c_{k}^{{rs}}$: Travel time of path *k* between OD pair (*r*, *s*)
$gc_{k}^{{rs}}$: Generalized travel cost of path *k* between OD pair (*r*, *s*)
*ρ*: On-time arrival probability
*p*_*r*_(·), *p*_*s*_(·), *p*_*a*_(·): Soft penalty functions
*θ*: The parameter in the soft penalty function
*ε*: The tolerance error

## Footnotes

1 The detailed explanation can be found in Proposition 1 of “Properties of the proposed model” section.

## Code availability

Code is available on request.
